# High Expression of Complement Component 5 (*C5*) at Tumor Site Associates with Superior Survival in Ewing's Sarcoma Family of Tumour Patients

**DOI:** 10.5402/2011/168712

**Published:** 2011-10-02

**Authors:** Suvi Savola, Arto Klami, Samuel Myllykangas, Cristina Manara, Katia Scotlandi, Piero Picci, Sakari Knuutila, Jukka Vakkila

**Affiliations:** ^1^Department of Pathology, Haartman Institute and HUSLAB, University of Helsinki and Helsinki University Central Hospital, 00014, Helsinki, Finland; ^2^Department of Information and Computer Science, Aalto University School of Science, 02015, Espoo, Finland; ^3^Laboratorio di Ricerca Oncologica, Istituti Ortopedici Rizzoli, 40136, Bologna, Italy; ^4^Hematology Research Unit, Helsinki University Central Hospital, P.O. Box 700, 00290, Helsinki, Finland

## Abstract

*Background*. Unlike in most adult-onset cancers, an association between typical paediatric neoplasms and inflammatory triggers is rare. We studied whether immune system-related genes are activated and have prognostic significance in Ewing's sarcoma family of tumors (ESFTs). *Method*. Data analysis was performed on gene expression profiles of 44 ESFT patients, 11 ESFT cell lines, and 18 normal skeletal muscle samples. Differential expression of 238 inflammation and 299 macrophage-related genes was analysed by *t*-test, and survival analysis was performed according to gene expression. *Results*. Inflammatory genes are activated in ESFT patient samples, as 38 of 238 (16%) inflammatory genes were upregulated (*P* < 0.001) when compared to cell lines. This inflammatory gene activation was characterized by significant enrichment of macrophage-related gene expression with 58 of 299 (19%) of genes upregulated (*P* < 0.001). High expression of complement component 5 (*C5*) correlated with better event-free (*P* = 0.01) and overall survival (*P* = 0.004) in a dose-dependent manner. C5 and its receptor C5aR1 expression was verified at protein level by immunohistochemistry on an independent ESFT tumour tissue microarray. *Conclusion*. Immune system-related gene activation is observed in ESFT patient samples, and prognostically significant inflammatory genes (*C5, JAK1*, and *IL8*) for ESFT were identified.

## 1. Introduction

Adult cancer is frequently preceded by a period of prolonged chronic inflammation caused by infectious microbial agents (Hepatitis B or C, human papillomavirus, Ebstein-Barr virus, and Helicobacter pylori) and other physical agents or irritants (repeated UV exposure, prolonged exposure to tobacco smoke, or asbestos fibers) [1]. Such preneoplastic inflammatory period may persist for several decades before a state of genomic instability and the full neoplastic phenotype is acquired. The role of the chronic inflammatory microenvironment as a causative factor is strongly supported also by findings that regular use of nonsteroidal anti-inflammatory drugs (NSAIDs) is associated with a reduced incidence of colorectal, breast, and gastric cancer [2–4]. On the contrary, an association between typical paediatric neoplasias (for example small round blue cell tumours) and inflammatory triggers is very rare. Whereas the expression of cyclooxygenase-2 (COX-2), an inducible enzyme active in inflammatory sites, has a tumour promoting activity in several adult cancers, studies in paediatric osteosarcoma, Ewing sarcoma, and rhabdomyosarcoma have failed to show any correlation between the activity of COX-2 and patient outcome [5]. 

The inflammatory preneoplastic period that is typical in adult but uncommon in paediatric cancers could result in qualitative and quantitative differences in tumour infiltrating leukocytes. This hypothesis was confirmed in our previous immunohistochemistry study in which we found that in adult tumours infiltrating leukocytes were composed of diverse leukocyte types, but in paediatric tumours, the infiltrating cells were predominantly macrophages, and dendritic cells were virtually absent [6]. The finding suggested that in typical paediatric tumours that evidently are not originating from chronic inflammation, the immune system may not have similarly significant role either in carcinogenesis or immune surveillance of tumours as in adult cancer [1]. 

Microarray-based gene expression profiling provides data about genes that are either over- or underexpressed in studied samples. Ewing sarcoma family of tumours (ESFTs) represent typical paediatric small-round blue cell tumours. We postulated that by comparing gene expression profiles of clinical ESFT tumour samples and Ewing sarcoma cell lines, we could reveal inflammatory gene signals derived from ESFT-associated immune component. In addition, gene expression differences between clinical ESFT samples and normal muscle tissues were determined to identify inflammatory genes that are functional in ESFT tumours but not in surrounding tissues. Prognostic significance of these findings was assessed by analysing cumulative outcome data of the patients. Our results suggest that high local production of complement component 5 (*C5)* and janus kinase 1 (*JAK1*) at tumour site associates with better survival of ESFT patients, whereas locally produced interleukin 8 (*IL8)* is detrimental.

## 2. Patients and Methods

### 2.1. Patients and Clinical Samples

The majority of ESFT patients were diagnosed with Ewing sarcoma (*n* = 32), ten with primitive neuroectodermal tumour (PNET), and two with Askin's tumour (Table 1). The patient material for study was taken prior to any treatment in 29 cases, and in 15 cases, chemotherapeutics, radiation therapy, and/or surgical treatment was applied before material was collected (see Table 1 for details). All patients were treated within controlled prospective trials [7, 8]. Of the 44 samples used in data analysis, 32 were primary tumours, five recurrences, and seven metastases. The mean age of the patients was 17.9 years, ranging from 4 to 34 years and the male to female ratio was 1.75 to 1. Median follow up was 44.8 months. For detailed data on patient material, see Table 1. This study was reviewed and approved by the Ethical Review Board of Helsinki University Central Hospital (decision no. 329 HUS/E0/05 and 328 HUS/W0/05). In order to ensure the anonymity of the patients, a generic sample code was given to each specimen, and all personal data was eliminated when subjected to this research.

### 2.2. Expression Data

Data analysis was performed on Affymetrix Human Genome U133 Plus 2.0 gene expression profiles of 44 ESFT patient samples previously published by Savola et al. [9], and original microarray data of these samples is available at gene expression omnibus (GEO) [10] with accession number GSE17679. Prior to expression analysis, patient material was checked to contain tumour tissue >75% using histological analysis. In brief, 5 *μ*g of total RNA was reverse transcribed to cDNA using One-Cycle cDNA Synthesis Kit (Affymetrix), according to the manufacturer's GeneChip One-Cycle Target Labeling-protocol. Biotin-labeling of antisense cRNA was carried out using IVT Labeling Kit (Affymetrix). The labeled and fragmented cRNA (15 *μ*g of each) was hybridized for 16 h at 45°C in a hybridization oven 640 (60 rpm). Washing and staining of the arrays with streptavidin-phycoerythrin (SAPE) was completed in a Fluidics Station 450 (Affymetrix). The arrays were then scanned using a confocal laser GeneChip Scanner 3000, and images were analyzed using GeneChip Operating Software (GCOS; Affymetrix, Sacramento, Calif, USA). Expression profiles (*n* = 11) of ESFT cell line samples (6647, IOR/BRZ, IOR/CAR, IOR/CLB, IOR/NGR, IOR/RCH, LAP35, RDES, SKES1, SKNMC, and TC71) were produced by the same method as described previously [9], and they are found at GEO with accession number GSE17679. Expression profiles (*n* = 18) of normal skeletal muscle tissue were selected from GEO with accession no. GSE6798 and GSE3526.

### 2.3. Annotated Gene Lists and Gene Expression Signatures

First, a collection of 238 genes putatively related to inflammation was constructed by literature review and included, for example, genes-encoding chemokines, chemokine receptors, cytokines, cytokine receptors, signalling molecules, and Toll-like receptors (see Supplemental data 1 which available online at: doi: 10.5402/2011/168712). This step was performed before looking at the ESFT expression data. Finally, a gene expression signature was derived from macrophages. A list of 299 genes expressed in macrophage cells was extracted from the SymAtlas [11], which contains Affymetrix U133 gene expression data from 122 normal cells and 90 cancer cell lines. There were 18 samples from the immune system-specific cell types of which two were CD14 positive cells. Macrophage-specific gene signature (*n* = 299) was extracted by filtering SymAtlas data according to the present/absent flags of MAS5 (Microarray Suite 5.0 statistical algorithm) preprocessed Affymetrix data. To be selected for inclusion in the macrophage specific gene list, probe sets were required to be present in both macrophage samples. In addition, selected probe sets were required to be absent in at least 50% of the samples of nonmacrophage immune system cells, 50% of normal cells and 50% of NCI 60 cell lines. 

### 2.4. Analysis of Differential Gene Expression and Inflammatory Response

All known protein coding genes were extracted from the NCBI38 data set in the Ensembl database [12], and a single Affymetrix probe set was chosen to represent each gene. Out of the probe sets annotated with a particular gene, the one with highest average expression level was chosen to represent that gene so that unique probe sets were given a priority (i.e., all genes that were annotated to match at least one unique probe set are represented by such probe sets). The purpose for taking protein coding genes as a base set instead of working directly at the Affymetrix probe set level was to remove potential bias caused by the manually constructed inflammation gene list consisting solely of genes known to code proteins. The macrophage list defined at the probe set level was transformed to the gene level using the Ensembl annotations. All genes that included any of the macrophage probe sets as a potential match were included to the list used in the data analysis, resulting in a list of 299 genes.

Differential expression was measured for each gene by a combination of two-sided *t*-test and fold change comparison. Two separate tests were conducted, one between the patient and cell line samples and another between the patient samples and muscle tissues. For both experiments, the corresponding gene expression collections were preprocessed with Robust Multi-Array Average (RMA) [13], and the *P* values of the *t*-test were corrected for multiple testing using the *q*-value procedure [14]. Genes with *q*-value below 0.01, indicating false discovery rate (FDR) of 1%, and fold change >1.6 (Ewing patient samples versus cell lines) or >2.15 (Ewing patient samples versus muscle tissue) were considered differentially expressed. The thresholds for the fold change were derived based on the expression levels of 14 housekeeping genes (*RPS13, RPS20, RPL30, RPL13A, RPL9, SRP14, RPL24, RPL22, RPS29, RPS16, RPL4, RPL6, OAZ1, *and* RPS12*) in the same collections, choosing the lowest fold change not exceeded by any of the housekeeping genes.

Fisher's exact test was used to study the association between inflammation and expression, the null hypothesis being that there is no connection between a gene being on the list of inflammation genes and having differential expression. Identical test was performed for the list of genes active in macrophage cells. 

### 2.5. Immunohistochemistry

Avidin-biotin-peroxidase procedure was used for immunostaining. Briefly, ESFT tumour tissue microarray sections were treated sequentially with xylene and ethanol to remove paraffin. Endogenous peroxidase activity was blocked by treatment with 3% hydrogen peroxide in methanol for 30 min at RT. No antigen retrieval was performed. To avoid background, a further blocking step with normal horse serum (Vector Laboratories, Burlingame, Calif, USA) was performed. *C5 *and *C5aR1* expression was analysed using anti-C5 goat antiserum (Quidel, San Diego, Calif; dilution 1 : 100) and anti-C5R1 rabbit polyclonal antibody (Abcam, Cambridge, UK; dilution 1 : 200) and on tissue microarray (TMA) containing ESFT patient samples in triplicates. Tissue sections were incubated with secondary biotinylated antimouse antibody and with an avidin-biotin-peroxidase complex (Vector Laboratories). The final reaction product was revealed by exposure to 0.03% diaminobenzidine (Sigma, St. Louis, Mo, USA) and nuclei were counterstained with Mayer's hematoxylin (Sigma). Patients were scored as negative or positive based on immunoreactivity of the respective antibody on tumour cells. For *C5aR1* and *C5* expression normal kidney and heart were, respectively, used as positive control.

### 2.6. Survival Analysis

For evaluating the prognostic value of each gene included in the list of nonmalignant (potential infiltrating immune/stromal) cell-derived (*n* = 10) and malignant (putatively ESFT) cell-derived genes (*n* = 22), we calculated its median expression value from RMA signal values (log2), and patients were labelled as having high expression or low expression relative to the median value. Patient survival analysis was then performed by Kaplan-Meier and Log Rank methods considering either event-free survival or overall survival.

## 3. Results

### 3.1. Inflammatory Genes Are Activated in ESFT Patient Samples and this Activation Is Characterized Primarily by Macrophage Gene Activation

**Figure 1:**
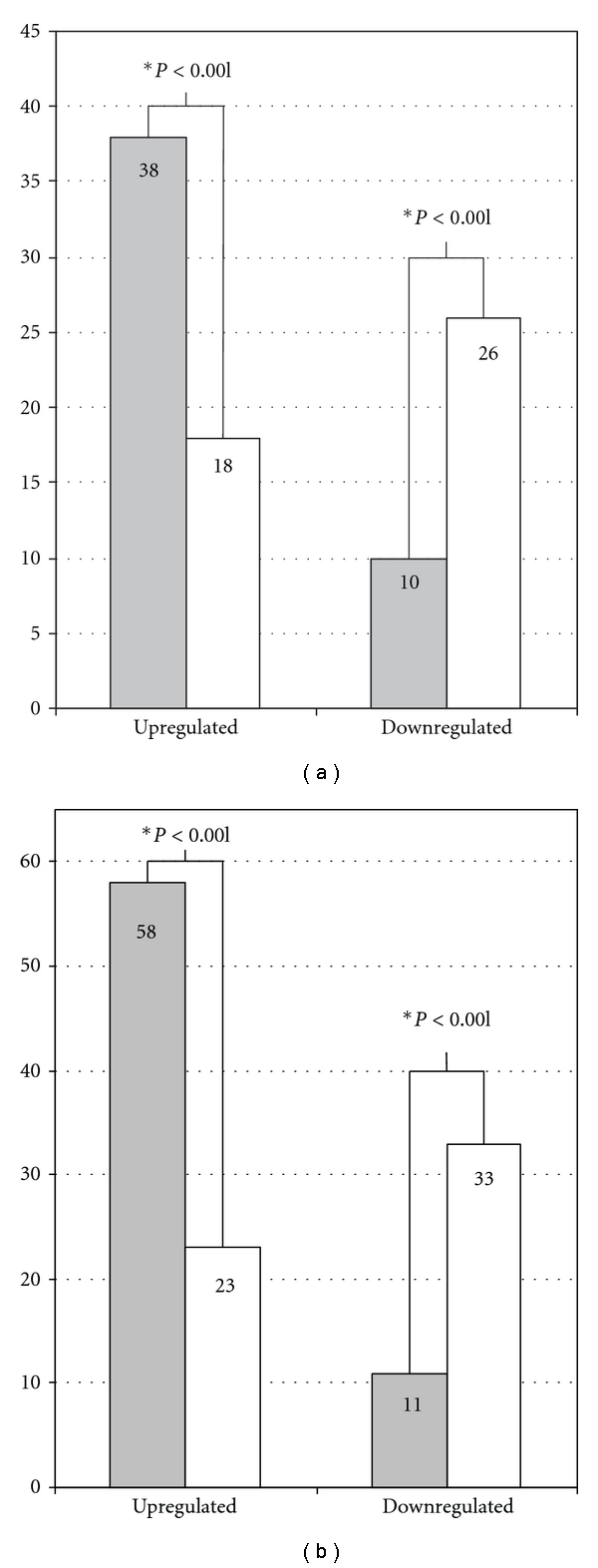
Differentially expressed inflammation and macrophage genes in Ewing sarcoma patient samples. (a) Differentially expressed inflammation genes and (b) macrophage genes by comparing Ewing patient samples (*n* = 44) to cell lines (*n* = 11). On *Y*-axis, the detected number of inflammatory genes and macrophage genes are marked with grey and the number of expected genes with white bar. The level of statistical significance is marked below the bars and a figure with ∗ indicates a statistically significant result.

Inflammatory signals deriving potentially from nonmalignant stromal cells or tumour- associated leukocytes were revealed by comparing ESFT patient samples to Ewing sarcoma cell lines. Our bioinformatic analysis detected that in ESFT patient samples, 38 of 238 (16%) inflammatory genes were significantly upregulated and 10 of 238 (4%) downregulated (thresholds: *q*-value <0.01 and fold change >1.6) (see Figure 1(a)). The number of expected upregulated and downregulated genes was 18 and 26, respectively. The number of verified upregulated inflammatory genes was statistically higher (*P* < 0.001) and the amount of downregulated inflammatory genes was statistically lower (*P* < 0.001) than expected in ESFT patient samples. Supplemental data 2 lists observed over- and underexpressed inflammation genes including their fold changes, *q*-values and *P* values when ESFT patient samples were compared to ESFT cell lines. Macrophage cell activity in ESFT tumour microenvironment was studied using *t*-test on 299 macrophage-related genes and performing a comparison between ESFT patient samples and cell lines. *t*-test showed that 58 of 299 (19%) macrophage genes were significantly upregulated and 11 of 299 (4%) were downregulated in ESFT samples (see Figure 1(b)). The number of expected upregulated macrophage genes was 23, and the number of downregulated genes was 33, which indicates that the amount of upregulated macrophage genes was statistically higher (*P* < 0.001) and the amount of downregulated macrophage genes was statistically lower (*P* < 0.001) in ESFT patient samples (Figure 1(b)). Thus, the inflammatory gene expression signature in ESFT patient samples is potentially described by macrophage gene activation. Supplemental data 3 lists over- and underexpressed macrophage genes including their fold changes, *q*-values and *P* values. 

### 3.2. Combining Data from ESFT Patient, Cell Line, and Normal Muscle Samples Reveals Potentially Important Inflammatory Genes under In Vivo Conditions

32 of 238 (13%) inflammatory genes were upregulated and 10 of 238 (4%) downregulated when ESFT patient samples were compared to normal skeletal muscle cell samples (thresholds: *q*-value <0.01 and fold change >2.15). Supplement 4 lists over- and underexpressed inflammation genes including their fold changes, *q*-values and *P* values. We postulated that these inflammatory genes distinguish ESFT tumour site from adjacent normal tissue that by large is composed of muscular tissues surrounding the tumour site. By combining expression data from ESFT patients, cell lines, and normal muscle tissue samples, we were able to create two separate lists of genes. The first list included 10 genes (see Table 2) and was composed of genes whose expression was upregulated both by comparing ESFT patient samples to cell lines and, additionally, ESFT patient samples to skeletal muscle tissue samples. These genes represent genes that are potentially derived from nonmalignant stromal cells or infiltrating immune cells and are distinguishing tumour site from surrounding normal tissue. A second list included 22 genes (see Table 3) and was composed of upregulated genes that were obtained by comparing ESFT patient samples to skeletal muscle tissue samples but were not upregulated by comparing ESFT patient samples with cell lines. These genes were assumed to represent inflammatory genes that distinguish ESFT tumours from surrounding normal tissues *in vivo*, but are derived from malignant component of tumour samples, that is, Ewing sarcoma cells.

### 3.3. High Expression of C5 in ESFT Is a Prognostic Factor for Favourable Clinical Outcome

**Figure 2:**
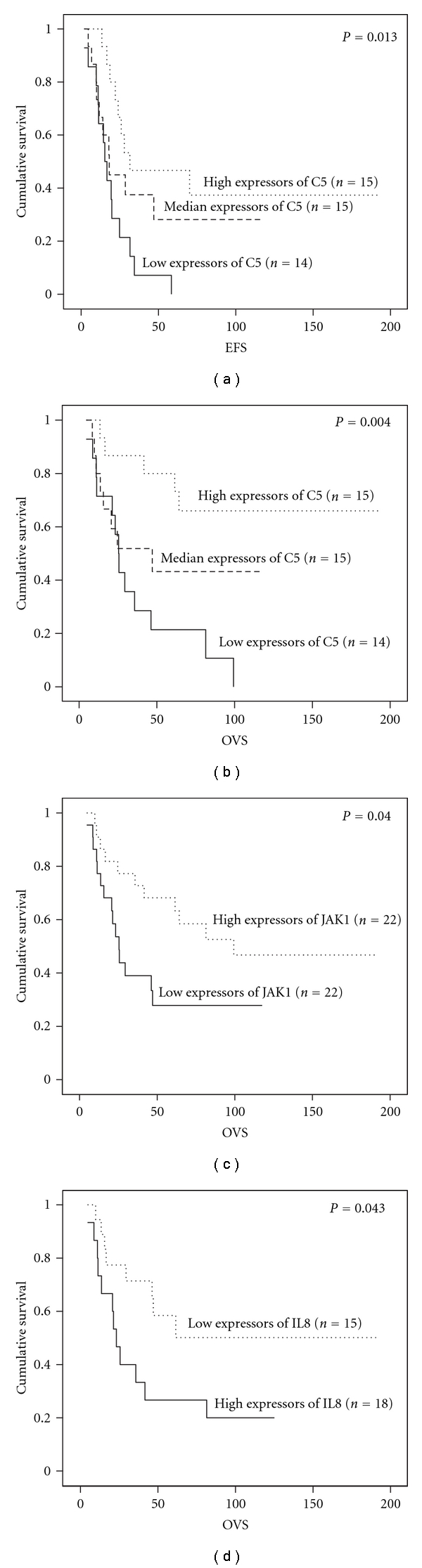
Correlation of inflammatory gene expression to ESFT patient survival. Kaplan-Meier analysis shows (a) event-free survival (EFS), (b) overall survival (OVS) of all ESFT patients (*n* = 44) according to *C5* expression, (c) overall survival of all Ewing sarcoma patients (*n* = 44) according to *JAK1* expression, and (d) overall survival of classical Ewing sarcoma patients (*n* = 33) according to *IL8* expression.

Next, we clarified, whether inflammation-associated genes (See Tables 2 and 3) are associated with patient prognosis. The best correlation between expression and patient survival was seen on complement component *C5* belonging to the genes that did not differ between patient samples and ESFT cell lines but whose expression was high at tumour site compared to adjacent normal tissue (see Table 3). High expression of *C5* associated with better event free survival (*P* = 0.01) and overall survival (*P* = 0.004) in a dose-dependent manner in Ewing sarcoma (*n* = 44) (see Figures 2(a) and 2(b)). In addition to *C5*, high expression of *JAK1* (see Table 3) correlated with favorable overall survival (*P* = 0.04) across all samples (Figure 2(c)). *IL8* was included in immune or nonmalignant stromal cell-derived gene list (Table 2) and high expression of *IL8* correlated with reduced overall survival (*P* = 0.04) in classical Ewing sarcoma (*n* = 33) (Figure 2(d)).

### 3.4. C5 and, Its Receptor, C5aR1 Expression at Protein Level is Detected in Ewing Sarcoma Cells of ESFT Patient Samples

**Figure 3:**
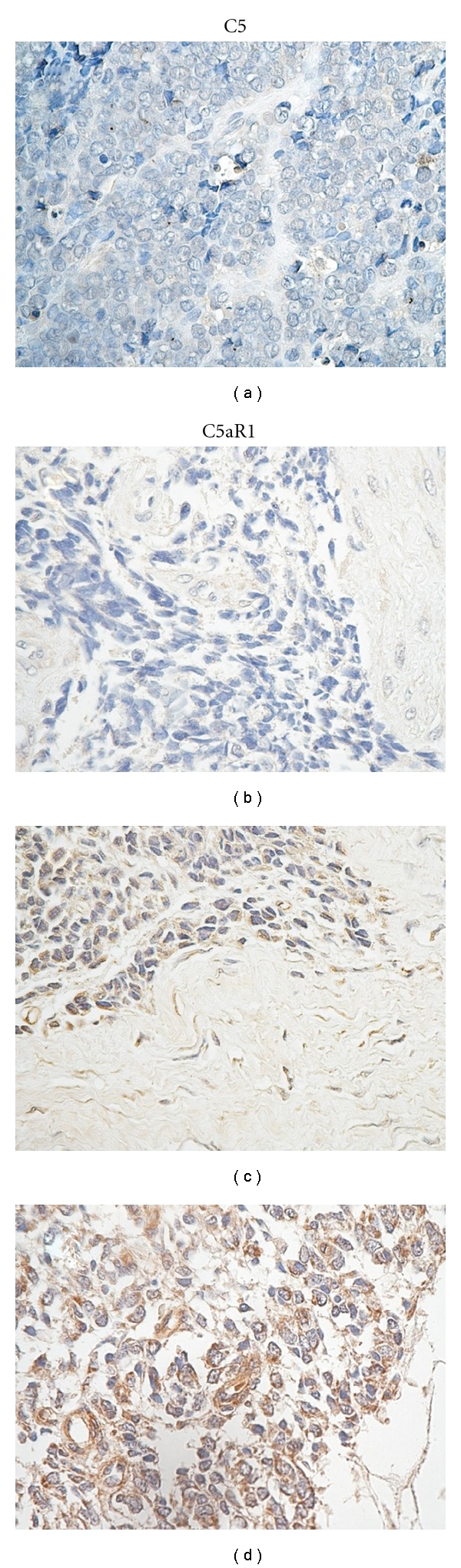
Immunohistochemical staining of C5 and C5aR1 on ESFT tissue microarray. (a) Negative staining of C5 in Ewing sarcoma cells; (b) Negative staining of C5aR1 in Ewing sarcoma cells; (c) Positive cytoplasmic staining of C5 in Ewing sarcoma cells; (d) Positive cytoplasmic and membrane staining of C5aR1 in Ewing sarcoma cells and weak positivity in matrix including fibroblasts. Original magnification ×400.

The results of the *C5 *gene expression were verified in an independent group of ESFT patients. In this context, we performed an immunohistochemical analysis on ESFT tissue microarray to validate the data on protein level and also to decipher whether ESFT cells express C5 and its receptor C5aR1. Of the 83 sections scored, 50 (60%) were positive, and 33 (40%) were negative for C5 protein expression. For C5 receptor, of the 255 sections scored with C5aR1 antibody, 142 (56%) were positive and 113 (44%) were negative for C5aR1 expression (Figure 3). As shown in Figures 3(c) and 3(d), malignant cells in tissue sections were positive for C5 and C5aR1, whereas the matrix stromal cells composed mainly of fibroblasts were mostly negative for C5 and C5aR1. However, weak positivity for C5aR1 in matrix was detected, as shown in Figure 3(d).

## 4. Discussion

Extensive research has shown that stroma and infiltrating cells within the tumour microenvironment play an important role in tumour biology [1, 15]. In this respect, our study was designed to elucidate the inflammatory microenvironment in ESFT using microarray-based expression analysis and bioinformatic tools. The expression analysis was performed on 44 ESFT patients, which is a substantial amount of samples in this rare tumour entity, and furthermore, the results concerning *C5* were validated on an independent set of ESFT samples on tumour tissue microarray by immunohistochemistry. The results suggest that in ESFT, inflammatory genes are activated and that the inflammatory component is characterized mainly by genes related to macrophages. In addition, the survival analysis showed that high local expression of *C5* and *JAK1* was beneficial for ESFT patient survival, whereas high expression of *IL8 *correlated with poor outcome.

In our previous study, we found that in paediatric solid tumours, including ESFTs, the predominant infiltrating cells were CD68+ macrophages, which accumulated in these tumours at the areas of necrosis [6]. Accordingly, in this study, a significant enrichment of macrophage-related gene expression was detected, suggesting that macrophages infiltrate ESFT site in vivo. This strengthens the results of previous studies also by others [16, 17]. Tumour-associated macrophages (TAMs) are key regulators between inflammation and cancer, and they are engaged in various steps of tumour progression for example by contributing tumour growth and angiogenesis by releasing growth factors and even by incurring immunosuppression [1, 18]. Macrophages are known to be active in phagocytosis of necrotic/apoptotic tumour tissue, but additionally, recent findings suggest that TAMs present in ESFTs might differentiate by RANKL- and TNF-*α*-dependent mechanism to osteoclasts, which in turn participate in the bone resorption as a pathogenic mechanism of tumour osteolysis [19]. 

ESFTs usually develop in bone; however, they may arise also from soft tissues in which case they are called extraosseous Ewing's sarcoma. Even, with a primary tumour that originates intraosseously, a soft-tissue component is frequently present owing to local invasion into adjacent muscular tissue through the cortical plate. We postulated that by comparing the inflammatory gene expression of ESFT samples with that of normal muscle samples, we could find genes that are distinguishing and characterizing the tumour site *in vivo* from surrounding normal (=muscular) tissue. These particular genes might involve, for example, chemokines, chemokine receptors, suppressive factors, or danger-associated molecular patterns (DAMPs). By identifying highly upregulated ESFT-associated inflammatory genes and connecting their expression levels with prognosis of patients, it might be possible to find new candidate genes for drug development. 

Our research strategy resulted a list of 32 ESFT-associated genes that were further separated into two groups: (a) nonmalignant inflammatory or stromal cell-derived genes (Table 2) and (b) the genes derived from malignant transformed cells within the tumour tissue (Table 3). The fold change was highest (28.3) for SPP1 (osteopontin) that is one of the major extracellular matrix proteins in bone and is produced by numerous cell types including osteoblasts and osteoclasts, but also by NK cells, T cells, macrophages, and cancer cells [20, 21]. High expression of SPP1 in tumour samples but not in cell lines (see Table 2) suggests that this gene is mostly expressed by stromal cells and other infiltrating cells within tumours but not by ESFT-cells themselves. In spite of its high expression, SPP1 (or some other highly expressed genes, e.g., CXCR4) did not correlate with patient survival, which could be explained by several ways. For example, if the majority of tumours are almost uniformly expressing some particular genes and related proteins, the influence of the variation in their expression level may not be easily detected. This may be the case also with chemokine receptor CXCR4, which was another gene that was strongly expressed in ESFT primary tumor samples. CXCR4 together with its cognate ligand CXCL12 (stromal cell-derived factor-1/SDF-1) is widely expressed in various different cancers, and it is somewhat surprising that its expression did not correlate with patient survival. It is also possible that high gene expression is not related with any such critical functions that have profound impact on patient survival. Thus, high gene expression does not necessarily mean that a gene or related protein is a good drug target nor does the lack of correlation between a gene, and patient survival implicates that the gene does not play a role in tumour progression *in vivo.*

The survival analysis showed that high expression of *IL8*, also known as *CXCL8*, associates with unfavourable survival in classical Ewing sarcoma of bone. IL8 is known to be a highly proinflammatory chemokine, which binds to CXCR1 and CXCR2. In the tumour microenvironment, IL8 has shown to contribute tumour growth and progression by enhancing tumour cell proliferation and survival, to promote angiogenesis by activating endothelial cells, and finally to facilitate metastasis by enhancing cell invasion and migration [22]. In this context, our results suggesting the prognostic significance of *IL8* and in ESFT are plausible and further strengthen the current view of *IL8* function in tumour biology. 

JAK1 and complement component C5 are important proteins involved with early steps of acute inflammation. JAK1 is a protein tyrosine kinase that mediates initial steps of interferon-*α*, -*β*, and -*γ* signal transduction. Interferons are produced at early steps of acute inflammation for example, by natural killer cells (NKs) and plasmacytoid dendritic cells (DCs) and one of their main roles is activation of tumour killing functions of macrophages. Type I interferons are also used in the treatment of various cancers in clinic. Our data indicates that high expression of *JAK1* correlates with favourable clinical outcome in ESFT, and it suggests that at least in some patients an advantageous acute inflammatory reaction is ongoing in tumour tissue. One should bear in mind, however, that mutations in *JAK1* resulting in its constitutive activation have been suggested to be the initial defects in several human cancers [23] and inhibition of JAK1 has been shown to induce apoptosis and to reduce tumour cell invasion in colorectal cancer cells [24]. 

To our knowledge, this paper is the first to show an association between locally produced *C5* expression and favourable patient survival in human cancers. The results on immunohistochemistry provided an independent verification of the gene expression analysis of *C5.* C5 is a part of the complement cascade system that initiates acute inflammatory responses. The activation of complement system results in the cleavage of C5 to its active subcomponents C5a and C5b. C5a is an anaphylatoxin, which is a powerful inflammatory mediator with chemotactic activity especially for neutrophils, but also for monocytes and macrophages [25]. On the other hand, C5b forms together with C6, C7, C8 and C9 a complement membrane attack complex (MAC), which ultimately leads to the lysis of the target cell. Even though the complement system has been conventionally considered to present a part of innate immune system, accumulating data suggests that complement system and C5a are able to activate cells involved in both innate and adaptive immunity [26]. Locally produced C5a did augment naive T cell survival and proliferation [27] and additionally enhanced T cell viability and expansion by suppressing antigen-induced programmed cell death [28]. Interestingly, in a previous study, C5a in a tumour microenvironment promoted tumour growth by activation of myeloid-derived suppressor cells [29]. It should be underlined, however, that this observation was made in a mouse model of cervical cancer. The observation of the expression of both *C5* and *C5aR1* in ESFT cells is very interesting and indicates that an autocrine loop might be present. C5a is a powerful proinflammatory factor and triggers release of DAMPs from target cells. Of interest, mesenchymal stem cells, from which ESFTs are suggested to be originating from, express C5aR1 and its activation by C5a cause prolonged ERK1/2 phosphorylation [30]. Further efforts should be taken on what C5 activation elicits on ESFT cells and which kind of downstream signalling pathways are triggered. Ultimately, the function of C5 in ESFT and other tumour types needs to be investigated more profoundly, and its role in tumour biology remains to be elucidated.

## 5. Conclusion

Our results suggest that even in cancers of noninflammatory origin like ESFTs an macrophage-enriched inflammatory microenvironment that distinguishes the tumour site from normal adjacent tissue is present at the time of clinically evident disease. Further, our results pointed out several prognostically significant inflammation-associated genes like *C5, JAK1* and *IL8*. To our knowledge, this paper is the first to show an association between locally produced *C5* expression and favourable patient survival in human cancers. Taken together, our findings suggest that immune cells and especially *C5* have an important role in controlling the progression of ESFT and that *C5* can be used as a clinically significant prognostic factor for ESFT patients.

## Supplementary Material

Supplemental data 1: List of inflammation related genes (n=238).
Supplemental data 2: Differentially expressed inflammation genes when ESFT patient samples (n=44) are compared to ESFT cell lines (n=11).
Supplemental data 3: Differentially expressed macrophage genes by comparing ESFT patient (n=44) samples to ESFT cell lines (n=33).
Supplemental data 4: Differentially expressed inflammation genes when ESFT patient samples (n=44) are compared to normal muscle samples (n=18).

## Figures and Tables

**Table 1:** Clinical data of 44 ESFT patient samples.

Sample code	Diagnosis	State	Age	Sex	EFS	OVS	Status	Treatment
R194	Ewing	Primary	32	F	11,7	15,6	Dead	−
R196	Ewing	Primary	5	M	14,0	20,7	Dead	−
R29	Ewing	Primary	21	M	4,6	4,6	Dead	−
R30	Ewing	Primary	26	F	28,6	48,9	NED	+
R33	Ewing	Primary	15	M	16,6	16,6	Dead	−
R34	Ewing	Primary	18	M	19,4	56,5	AWD	+
R35	Ewing	Primary	16	F	10,0	13,6	Dead	+
R37	Ewing	Primary	11	M	9,8	9,8	Dead	+
R38	Ewing	Primary	23	M	11,3	11,3	Dead	−
R39	Ewing	Primary	16	F	47,0	47,0	Dead	−
R40	Ewing	Primary	24	M	18,2	117,3	NED	−
R41	Ewing	Primary	15	M	125,1	125,1	NED	−
R42	Ewing	Primary	18	M	192,2	192,2	NED	−
R43	Ewing	Recurrence	17	F	24,8	35,7	Dead	+
R44	Ewing	Recurrence	30	F	19,8	25,6	Dead	+
R45	Askin	Recurrence	15	M	17,9	24,6	Dead	+
R46	PNET	Primary	7	F	22,1	64,2	Dead	−
R48	Ewing	Metastasis	17	M	31,6	46,2	Dead	+
R49	Ewing	Metastasis	34	M	18,6	126,2	NED	+
R50	PNET	Metastasis	16	M	58,3	99,3	Dead	−
R51	Askin	Primary	24	M	4,6	10,9	Dead	+
R52	Ewing	Recurrence	22	F	14,4	23,2	Dead	+
R53	Ewing	Primary	19	F	87,7	87,7	NED	−
R54	Ewing	Recurrence	22	M	25,9	61,5	Dead	+
R55	PNET	Metastasis	16	F	6,8	8,5	Dead	−
R57	Ewing	Primary	11	M	2,0	8,7	Dead	−
R58	Ewing	Primary	22	M	13,4	13,4	Dead	−
R60	Ewing	Primary	12	M	121,5	121,5	NED	−
R61	Ewing	Primary	15	M	16,2	16,2	NED	−
R62	Ewing	Primary	16	M	117,6	117,6	NED	−
R63	Ewing	Primary	13	M	43,4	43,4	NED	−
R64	Ewing	Primary	25	M	15,3	21,3	Dead	−
R65	Ewing	Primary	20	F	11,0	11,0	Dead	−
R67	Ewing	Primary	14	M	16,7	29,4	Dead	−
R69	Ewing	Primary	9	F	62,6	62,6	NED	−
R72	PNET	Primary	32	F	31,5	117,7	NED	−
R74	PNET	Primary	27	M	110,4	110,4	NED	−
R75	PNET	Primary	14	M	9,8	25,4	Dead	−
R78	PNET	Primary	11	F	69,5	69,5	NED	−
R79	PNET	Primary	21	M	127,1	127,1	NED	−
R80	PNET	Primary	8	F	23,9	68,5	NED	−
R81	Ewing	Metastasis	24	M	34,4	81,4	Dead	+
R83	PNET	Metastasis	4	F	70,1	129,1	NED	+
R84	Ewing	Metastasis	9	M	27,9	41,6	Dead	+

Mean			17,9		40,1	57,0		

Abbreviations: EFS: event-free survival; OVS: overall survival; NED: no evidence of disease; AWD: alive with disease; −: treatment not received before biopsy: +: treatment received before biopsy.

**Table 2:** The list of genes deriving from infiltrating immune or nonmalignant stromal cells (*n* = 10): The differences in gene expression between patient tumour sample and cell lines or between patient tumour sample and normal muscle tissue.

Gene name	Comparison	Log ratio	Fold change	*q*-value	*P* value
*SPP1 *	versus cell lines	5,608	48,757	1,22*E*-17	2,62*E*-20
	versus muscle	4,823	28,308	9,28*E*-19	7.30*E*-19

*CXCR4*	versus cell lines	3,870	14,621	2,00*E*-04	7,55*E*-05
	versus muscle	3,700	12,999	9,52*E*-22	4,67*E*-22

*CD14*	versus cell lines	3,089	8,509	8,47*E*-15	4,10*E*-17
	versus muscle	2,431	5,391	6,04*E*-15	8,62*E*-15

*FOS*	versus cell lines	2,984	7,911	5,19*E*-05	1,44*E*-05
	versus muscle	2,346	5,086	1,37*E*-04	9,04*E*-04

*TNFAIP3*	versus cell lines	2,065	4,186	3,65*E*-05	9,29*E*-06
	versus muscle	1,411	2,659	1,46*E*-09	4,86*E*-09

*SOCS3*	versus cell lines	1,940	3,837	1,30*E*-08	6,82*E*-10
	versus muscle	1,793	3,464	6,81*E*-09	2,50*E*-08

*HSPA6*	versus cell lines	1,584	2,998	1,03*E*-07	7,79*E*-09
	versus muscle	1,353	2,554	8,41*E*-08	3,65*E*-07

*IL8*	versus cell lines	1,368	2,582	1,55*E*-03	9,18*E*-04
	versus muscle	1,500	2,828	1,02*E*-07	4,49*E*-07

*IL23A*	versus cell lines	1,259	2,393	1,13*E*-05	2,20*E*-06
	versus muscle	1,336	2,525	9,77*E*-14	1,70*E*-13

*STAT2*	versus cell lines	1,259	2,393	1,13*E*-05	2,20*E*-06
	versus muscle	1,336	2,525	9,77*E*-14	1,70*E*-13

**Table 3:** The list of malignant cell derived inflammatory genes (*n* = 22): The differences in gene expression between patient tumour sample and normal muscle tissue.

Gene name	Log ratio	Fold change	*q*-value	*P* value
*LY96*	4,465	22,085	1,26*E*-27	2,07*E*-28
*MIF*	3,843	14,352	1,01*E*-32	5,59*E*-34
*JAK1*	2,961	7,786	7,06*E*-24	2,41*E*-24
*MAPK7*	2,790	6,917	7,33*E*-17	7,77*E*-17
*PGLYRP2*	2,589	6,017	9,29*E*-15	1,37*E*-14
*ERLIN1*	2,235	4,709	5,16*E*-16	6,23*E*-16
*PELI1*	2,117	4,337	2,33*E*-14	3,65*E*-14
*SOCS4*	2,053	4,149	7,78*E*-15	1,13*E*-14
*TRAF5*	1,984	3,955	4,29*E*-17	4,38*E*-17
*IRF3*	1,889	3,703	3,54*E*-14	5,73*E*-14
*MYD88*	1,846	3,596	1,07*E*-17	9,93*E*-18
*SOCS2*	1,801	3,485	3,21*E*-11	8,29*E*-11
*STAT6*	1,656	3,151	1,93*E*-13	3,52*E*-13
*HMGB1*	1,585	2,999	8,87*E*-13	1,79*E*-12
*MAPK1*	1,447	2,727	7,10*E*-13	1,40*E*-12
*IL10RB*	1,278	2,426	2,16*E*-11	5,42*E*-11
*IKBKB*	1,199	2,296	7,47*E*-08	3,21*E*-07
*PELI2*	1,191	2,283	9,01*E*-09	3,37*E*-08
*C5*	1,185	2,273	2,48*E*-08	9,92*E*-08
*MAP4K4*	1,182	2,268	2,69*E*-11	6,85*E*-11
*STAT1*	1,139	2,202	4,11*E*-09	1,46*E*-08
*FOXP3*	1,112	2,161	6,34*E*-14	1,07*E*-13
