# Supplementary material for: High Expression of Complement Component 5 (C5) at Tumor Site Associates with Superior Survival in Ewing's Sarcoma Family of Tumour Patients
Source: ISRN Oncol. 2011 Oct 2;2011:168712. doi: 10.5402/2011/168712 (PMC3196920; doi:10.5402/2011/168712)
Supplement: Supplementary file 1 — Supplemental data 1: List of inflammation related genes (n=238). Supplemental data 2: Differentially expressed inflammation genes when ESFT patient samples (n=44) are compared to ESFT cell lines (n=11). Supplemental data 3: Differentially expressed macrophage genes by comparing ESFT patient (n=44) samples to ESFT cell lines (n=33). Supplemental data 4: Differentially expressed inflammation genes when ESFT patient samples (n=44) are compared to normal muscle samples (n=18). [file 168712.f1.pdf]

**Gene name**	**ensembl.gene.ID**	**AFFY.HG.U133.PLUS.2 probe**	**Chr.**	**Band**
ABCF1	ENSG00000204574	200045_at	6	p21.33
BCL6	ENSG00000113916	203140_at	3	q27.3
BLR1	ENSG00000160683	227616_at	11	q23.3
BTK	ENSG00000010671	205504_at	X	q22.1
C14orf21	ENSG00000196943	225514_at	14	q11.2
C5	ENSG00000106804	205500_at	9	q33.2
C9orf47	ENSG00000186354	1564274_at	9	q22.2
CCL1	ENSG00000108702	207533_at	17	q12
CCL11	ENSG00000172156	210133_at	17	q12
CCL13	ENSG00000181374	216714_at	17	q12
CCL15	ENSG00000161574	205392_s_at	17	q12
CCL16	ENSG00000161573	207354_at	17	q12
CCL17	ENSG00000102970	207900_at	16	q13
CCL18	ENSG00000006074	209924_at	17	q12
CCL19	ENSG00000172724	210072_at	9	p13.3
CCL2	ENSG00000108691	216598_s_at	17	q12
CCL20	ENSG00000115009	205476_at	2	q36.3
CCL21	ENSG00000137077	204606_at	9	p13.3
CCL22	ENSG00000102962	207861_at	16	q13
CCL23	ENSG00000167236	241161_at	17	q12
CCL24	ENSG00000106178	221463_at	7	q11.23
CCL25	ENSG00000131142	206988_at	19	p13.2
CCL26	ENSG00000006606	223710_at	7	q11.23
CCL3	ENSG00000006075	205114_s_at	17	q12
CCL4L2	ENSG00000129277	204103_at	17	q12
CCL5	ENSG00000161570	204655_at	17	q12
CCL7	ENSG00000108688	208075_s_at	17	q12
CCL8	ENSG00000108700	214038_at	17	q12
CCR1	ENSG00000163823	205098_at	3	p21.31
CCR2	ENSG00000121807	206978_at	3	p21.31
CCR3	ENSG00000183625	208304_at	3	p21.31
CCR4	ENSG00000183813	208376_at	3	p23
CCR5	ENSG00000160791	206991_s_at	3	p21.31
CCR6	ENSG00000112486	206983_at	6	q27
CCR7	ENSG00000126353	206337_at	17	q21.2
CCR8	ENSG00000179934	208059_at	3	p22.2
CCR9	ENSG00000173585	207445_s_at	3	p21.31
CD14	ENSG00000170458	201743_at	5	q31.3
CD180	ENSG00000134061	206206_at	5	q12.3
CD40	ENSG00000101017	35150_at	20	q13.12
CD40LG	ENSG00000102245	207892_at	X	q26.3
CEBPB	ENSG00000172216	212501_at	20	q13.13
CLEC4E	ENSG00000166523	219859_at	12	p13.31
CRP	ENSG00000132693	205753_at	1	q23.2
CSF2	ENSG00000164400	210228_at	5	q23.3
CSF3	ENSG00000108342	207442_at	17	q21.1
CTLA4	ENSG00000163599	234895_at	2	q33.2
CX3CL1	ENSG00000006210	203687_at	16	q13
CX3CR1	ENSG00000168329	205898_at	3	p22.2
CXCL10	ENSG00000169245	204533_at	4	q21.1
CXCL11	ENSG00000169248	210163_at	4	q21.1
CXCL12	ENSG00000107562	203666_at	10	q11.21
CXCL13	ENSG00000156234	205242_at	4	q21.1
CXCL14	ENSG00000145824	222484_s_at	5	q31.1
CXCL2	ENSG00000081041	209774_x_at	4	q13.3

CXCL3	ENSG00000163734	207850_at	4	q13.3
CXCL5	ENSG00000163735	214974_x_at	4	q13.3
CXCL6	ENSG00000124875	206336_at	4	q13.3
CXCL9	ENSG00000138755	203915_at	4	q21.1
CXCR4	ENSG00000121966	217028_at	2	q21.3
ELK1	ENSG00000126767	203617_x_at	X	p11.23
ERLIN1	ENSG00000107566	202441_at	10	q24.31
FOS	ENSG00000170345	209189_at	14	q24.3
FOXP3	ENSG00000049768	206016_at	X	p11.23
GPC1	ENSG00000063660	202756_s_at	2	q37.3
GZMB	ENSG00000100453	210164_at	14	q11.2
HMGB1	ENSG00000189403	224731_at	13	q12.3
HRAS	ENSG00000174775	212983_at	11	p15.5
HSP71_HUMAN	ENSG00000204389	200799_at	6	p21.33
HSPA4	ENSG00000170606	208814_at	5	q31.1
HSPA6	ENSG00000173110	213418_at	1	q23.3
HSPD1	ENSG00000144381	243372_at	2	q33.1
ICBR_HUMAN	ENSG00000118168	231733_at	11	q22.3
IFNA2	ENSG00000188379	211338_at	9	p21.3
IFNB1	ENSG00000171855	208173_at	9	p21.3
IFNG	ENSG00000111537	210354_at	12	q15
IKBKB	ENSG00000104365	209341_s_at	8	p11.21
IKBKG	ENSG00000073009	36004_at	X	q28
IL10	ENSG00000136634	207433_at	1	q32.2
IL10RA	ENSG00000110324	204912_at	11	q23.3
IL10RB	ENSG00000159113	209575_at	21	q22.11
IL11	ENSG00000095752	206924_at	19	q13.42
IL11RA	ENSG00000137070	204773_at	9	p13.3
IL12A	ENSG00000168811	207160_at	3	q25.33
IL12B	ENSG00000113302	207901_at	5	q33.3
IL12RB1	ENSG00000096996	239522_at	19	p13.11
IL12RB2	ENSG00000081985	206999_at	1	p31.2
IL13	ENSG00000169194	207844_at	5	q23.3
IL13RA1	ENSG00000131724	201887_at	X	q24
IL13RA2	ENSG00000123496	206172_at	X	q23
IL15	ENSG00000164136	205992_s_at	4	q31.21
IL15RA	ENSG00000134470	207375_s_at	10	p15.1
IL16	ENSG00000172349	1555016_at	15	q25.1
IL17A	ENSG00000112115	208402_at	6	p12.2
IL17C	ENSG00000124391	224079_at	16	q24.3
IL17RA	ENSG00000177663	205707_at	22	q11.1
IL18	ENSG00000150782	206295_at	11	q23.1
IL18R1	ENSG00000115604	206618_at	2	q11.2
IL19	ENSG00000142224	220745_at	1	q32.2
IL1A	ENSG00000115008	210118_s_at	2	q13
IL1B	ENSG00000125538	205067_at	2	q13
IL1R1	ENSG00000115594	202948_at	2	q11.2
IL1R2	ENSG00000115590	205403_at	2	q11.2
IL1RN	ENSG00000136689	216245_at	2	q13
IL2	ENSG00000109471	207849_at	4	q27
IL20	ENSG00000162891	224071_at	1	q32.2
IL21	ENSG00000138684	221271_at	4	q27
IL22	ENSG00000127318	222974_at	12	q15
IL23A	ENSG00000110944	225636_at	12	q13.3
IL23R	ENSG00000162594	1561853_a_at	1	p31.2
IL24	ENSG00000162892	206569_at	1	q32.2

IL26	ENSG00000111536	221111_at	12	q15
IL27	ENSG00000197272	1552995_at	16	p11.2
IL29	ENSG00000182393	1552917_at	19	q13.2
IL2RA	ENSG00000134460	206341_at	10	p15.1
IL2RB	ENSG00000100385	205291_at	22	q13.1
IL2RG	ENSG00000147168	204116_at	X	q13.1
IL3	ENSG00000164399	207906_at	5	q23.3
IL32	ENSG00000008517	203828_s_at	16	p13.3
IL33	ENSG00000137033	209821_at	9	p24.1
IL4	ENSG00000113520	207538_at	5	q23.3
IL5	ENSG00000113525	207952_at	5	q23.3
IL5RA	ENSG00000091181	207902_at	3	p26.2
IL6	ENSG00000136244	205207_at	7	p15.3
IL6R	ENSG00000160712	226333_at	1	q22
IL6ST	ENSG00000134352	212195_at	5	q11.2
IL8	ENSG00000169429	202859_x_at	4	q13.3
IL8RA	ENSG00000163464	207094_at	2	q35
IL8RB	ENSG00000180871	207008_at	2	q35
IL9	ENSG00000145839	208193_at	5	q31.1
INDO	ENSG00000131203	210029_at	8	p11.22
IRF1	ENSG00000125347	202531_at	5	q23.3
IRF3	ENSG00000126456	202621_at	19	q13.33
IRF7	ENSG00000185507	234952_s_at	11	p15.5
JAK1	ENSG00000162434	201648_at	1	p31.3
JAK2	ENSG00000096968	205841_at	9	p24.1
JAK3	ENSG00000105639	227677_at	19	p13.11
JUN	ENSG00000177606	213281_at	1	p32.2
LTA	ENSG00000204496	206975_at	6	p21.33
LTB	ENSG00000204487	207339_s_at	6	p21.33
LY86	ENSG00000112799	205859_at	6	p25.1
LY96	ENSG00000154589	206584_at	8	q21.11
MAL	ENSG00000172005	204777_s_at	2	q11.1
MAP2K1IP1	ENSG00000109270	217971_at	4	q23
MAP2K3	ENSG00000034152	215499_at	17	p11.2
MAP2K4	ENSG00000065559	203266_s_at	17	p12
MAP2K6	ENSG00000108984	205699_at	17	q24.3
MAP3K1	ENSG00000095015	225927_at	5	q11.2
MAP3K14	ENSG00000006062	205192_at	17	q21.31
MAP4K4	ENSG00000071054	222547_at	2	q11.2
MAPK1	ENSG00000100030	224621_at	22	q11.21
MAPK10	ENSG00000109339	204813_at	4	q21.23
MAPK11	ENSG00000185386	211500_at	22	q13.33
MAPK12	ENSG00000188130	206106_at	22	q13.33
MAPK13	ENSG00000156711	210058_at	6	p21.31
MAPK14	ENSG00000112062	202530_at	6	p21.31
MAPK3	ENSG00000102882	212046_x_at	16	p11.2
MAPK6	ENSG00000069956	243831_at	15	q21.2
MAPK7	ENSG00000166484	212713_at	17	p11.2
MAPK8	ENSG00000107643	226048_at	10	q11.22
MAPK8IP3	ENSG00000138834	218112_at	16	p13.3
MAPK9	ENSG00000050748	203218_at	5	q35.3
MBTPS1	ENSG00000140943	201620_at	16	q23.3
MDM2	ENSG00000135679	205385_at	12	q15
MIF	ENSG00000099964	217871_s_at	22	q11.23
MYD88	ENSG00000172936	209124_at	3	p22.3
NFKB1	ENSG00000109320	209239_at	4	q24

NFKB2	ENSG00000077150	209636_at	10	q24.32
NFKBIA	ENSG00000100906	231699_at	14	q13.2
NFKBIB	ENSG00000104825	228388_at	19	q13.2
NFKBIE	ENSG00000146232	203927_at	6	p21.1
NFKBIL2	ENSG00000160949	1558329_at	8	q24.3
NFRKB	ENSG00000170322	213028_at	11	q24.3
NM_005007	ENSG00000204498	209973_at	6	p21.33
PDCD1	ENSG00000188389	207634_at	2	q37.3
PDCD1LG2	ENSG00000197646	224399_at	9	p24.1
PELI1	ENSG00000197329	218319_at	2	p14
PELI2	ENSG00000139946	219132_at	14	q22.3
PF4	ENSG00000163737	206390_x_at	4	q13.3
PGLYRP1	ENSG00000008438	228472_at	19	q13.32
PGLYRP2	ENSG00000161031	242817_at	19	p13.12
PGLYRP3	ENSG00000159527	1553059_at	1	q21.3
PGLYRP4	ENSG00000163218	220944_at	1	q21.3
PRF1	ENSG00000180644	214617_at	10	q22.1
PTGES	ENSG00000148344	207388_s_at	9	q34.11
PTGS2	ENSG00000073756	204748_at	1	q31.1
REL	ENSG00000162924	206035_at	2	p16.1
RELA	ENSG00000173039	230202_at	11	q13.1
RELB	ENSG00000104856	205205_at	19	q13.32
RIPK2	ENSG00000104312	209544_at	8	q21.3
SARM1	ENSG00000004139	213257_at	17	q11.2
SCYE1	ENSG00000164022	202541_at	4	q24
SIGIRR	ENSG00000185187	52940_at	11	p15.5
SLA	ENSG00000155926	203761_at	8	q24.22
SOCS1	ENSG00000185338	213337_s_at	16	p13.13
SOCS2	ENSG00000120833	203373_at	12	q22
SOCS3	ENSG00000184557	227697_at	17	q25.3
SOCS4	ENSG00000180008	226178_at	14	q22.2
SOCS5	ENSG00000171150	208127_s_at	2	p21
SOCS7	ENSG00000174111	214015_at	17	q12
SPP1	ENSG00000118785	209875_s_at	4	q22.1
STAT1	ENSG00000115415	AFFX-HUMISGF3A/M97935_3_at	2	q32.2
STAT2	ENSG00000170581	225636_at	12	q13.3
STAT3	ENSG00000168610	208991_at	17	q21.2
STAT4	ENSG00000138378	206118_at	2	q32.3
STAT5A	ENSG00000126561	225289_at	17	q21.2
STAT5B	ENSG00000173757	212549_at	17	q21.2
STAT6	ENSG00000166888	212803_at	12	q13.3
TBK1	ENSG00000183735	218520_at	12	q14.2
TICAM1	ENSG00000127666	213191_at	19	p13.3
TIRAP	ENSG00000150455	1554091_a_at	11	q24.2
TLR1	ENSG00000174125	210176_at	4	p14
TLR10	ENSG00000174123	223751_x_at	4	p14
TLR2	ENSG00000137462	204924_at	4	q31.3
TLR3	ENSG00000164342	239587_at	4	q35.1
TLR4	ENSG00000136869	1552798_a_at	9	q33.1
TLR5	ENSG00000187554	210166_at	1	q42.11
TLR6	ENSG00000174130	207446_at	4	p14
TLR7	ENSG00000196664	220146_at	X	p22.2
TLR8	ENSG00000101916	229560_at	X	p22.2
TLR9	ENSG00000173366	202009_at	3	p21.2
TMED7	ENSG00000134970	228234_at	5	q22.3
TNF	ENSG00000204490	207113_s_at	6	p21.33

TNFAIP3	ENSG00000118503	202644_s_at	6	q23.3
TNFRSF1A	ENSG00000067182	207643_s_at	12	p13.31
TNFRSF1B	ENSG00000028137	203508_at	1	p36.22
TOLLIP	ENSG00000078902	217930_s_at	11	p15.5
TRADD	ENSG00000102871	1729_at	16	q22.1
TRAF1	ENSG00000056558	205599_at	9	q33.2
TRAF2	ENSG00000127191	204413_at	9	q34.3
TRAF3	ENSG00000131323	208315_x_at	14	q32.32
TRAF4	ENSG00000076604	202871_at	17	q11.2
TRAF5	ENSG00000082512	204352_at	1	q32.3
TRAF6	ENSG00000175104	205558_at	11	p12
TYK2	ENSG00000105397	205546_s_at	19	p13.2
VEGFA	ENSG00000112715	210512_s_at	6	p21.1
XCL1	ENSG00000143184	206365_at	1	q24.2
XCR1	ENSG00000173578	221468_at	3	p21.31
