# Supplementary material for: High Expression of Complement Component 5 (C5) at Tumor Site Associates with Superior Survival in Ewing's Sarcoma Family of Tumour Patients
Source: ISRN Oncol. 2011 Oct 2;2011:168712. doi: 10.5402/2011/168712 (PMC3196920; doi:10.5402/2011/168712)
Supplement: Supplementary file 2 [file 168712.f2.pdf]

Gene name	log ratio	fold change	q-value	p-value
ABCF1	-1.219548258	0.429417157941395	9.60348988743953e-07	1.08259664663728e-07
NFKBIL2	-1.069848708	0.476368951934966	0.000178195187664682	6.56547703557531e-05
HRAS	-1.061205094	0.479231586721493	0.000363406389049934	0.000156715253953512
TBK1	-0.947965251	0.518363035619531	0.000213247411440672	8.17510572521873e-05
SCYE1	-0.915419362	0.530189733074477	0.000697624652753544	0.000345120978960035
MAPK9	-0.899695788	0.535999742075226	9.0103396901448e-05	2.84242441238312e-05
MAP4K4	-0.845553648	0.556497206934316	0.003284363373176	0.0023435576034137 5
TRAF4	-0.767019366	0.587630275959442	0.000434606001732118	0.000195016644049202
TOLLIP	-0.763921173	0.588893570503257	0.00195291916286927	0.001222715156269
MAPK8IP3	-0.708789711	0.611833195507965	0.00322862394687381	0.00229229833336028
MAP3K14	0.71457584	1.64100068408167	6.50269369154767e-06	1.12108604529558e-06
CCL4L2	0.77771583	1.71441434895816	1.52417252422269e-05	3.16447141466511e-06
IRF1	0.825083894	1.77163805777994	4.08704978129276e-05	1.08204766548876e-05
CCL8	0.825937638	1.77268677079254	0.000115940842681576	3.84926042810059e-05
MAPK13	0.84854168	1.80067982365829	0.0028869864314132	0.0019996594740754
MAP2K3	0.851423215	1.80427996520346	2.12514067269525e-06	2.89063638583676e-07
TMED7	0.873593592	1.83222107900644	0.00660038605043878	0.00543530941669571
CX3CR1	0.876212526	1.83555014226663	3.04468018408972e-07	2.77172476273156e-08
CXCL2	0.895473828	1.86022074165531	0.000406566773466278	0.000180084042258662
TNFRSF1B	0.911271662	1.88070251175614	5.85996116731569e-08	3.87843373990012e-09
CCL15	0.931139319	1.90678121731926	3.70801996763621e-07	3.46263812846755e-08
JAK3	0.985115133	1.9794712764392	0.000419965026685248	0.000186696306849893
CCL3	1.076709616	2.10922005045948	2.73070369237112e-05	6.47728357491868e-06
LY86	1.081809683	2.11668954289018	1.93670587444289e-06	2.53654387761716e-07
CXCL9	1.083360153	2.11896557928029	8.91167718385805e-05	2.80890341482473e-05
TNFRSF1A	1.134755594	2.19581360541828	0.00114796298826155	0.000630270555421187
JUN	1.16650071	2.24466587252631	1.65957245049474e-08	8.9933400588381e-10
CCL5	1.214390037	2.32042655879354	4.70425508817401e-06	7.67504887037805e-07
IL32	1.253198563	2.38369320206793	0.00156259151032401	0.000928725739788146
IL23A	1.258681052	2.39276887882525	1.13117806850915e-05	2.19643280774826e-06
STAT2	1.258681052	2.39276887882525	1.13117806850915e-05	2.19643280774826e-06
IL13RA1	1.354689185	2.55742013177808	0.00072174787636514	0.000358898938643942
IL2RG	1.362333262	2.57100650868046	8.30106904727871e-06	1.50914326667321e-06
IL8	1.368382696	2.58180975349167	0.00154873912507317	0.000918201760626705
CCL18	1.376291042	2.59600118457471	1.85619041895636e-05	4.03850659484160e-06
IL6ST	1.416477577	2.66932982506874	3.15800935800221e-05	7.77113144336081e-06
SLA	1.426910068	2.68870238005981	1.69349736608532e-10	4.07621712285705e-12
IL10RA	1.4281038	2.69092801809946	4.03242505012149e-08	2.46173845204860e-09
HSPA6	1.583856246	2.99770048958419	1.03225940298395e-07	7.787023070017e-09
SOCS3	1.939878769	3.83673406046988	1.30476294450875e-08	6.82497347707438e-10
TNFAIP3	2.065487408	4.18575365918698	3.65412373557777e-05	9.2916861209129e-06
CXCL14	2.074813071	4.21289824754368	7.82805206248583e-06	1.40844457048084e-06
IL1R1	2.650553799	6.27908263694852	3.03537767496686e-12	3.67345731279806e-14
HSP71_HUMAN	2.831145283	7.11638855020679	0.000954044010140487	0.000499891378293691
FOS	2.983897962	7.91120776977933	5.18638913415677e-05	1.43944323889994e-05
CD14	3.088907546	8.50851610612987	8.47499718898979e-15	4.10262494273335e-17
CXCR4	3.869939387	14.620688922406	0.000199639169073328	7.54795028480725e-05
SPP1	5.607526351	48.7566247258494	1.21715839214518e-17	2.61870654088205e-20
