# Supplementary material for: High Expression of Complement Component 5 (C5) at Tumor Site Associates with Superior Survival in Ewing's Sarcoma Family of Tumour Patients
Source: ISRN Oncol. 2011 Oct 2;2011:168712. doi: 10.5402/2011/168712 (PMC3196920; doi:10.5402/2011/168712)
Supplement: Supplementary file 3 [file 168712.f3.pdf]

Gene name	log ratio	fold change	q-value	p-value
DKC1	-1.397219295	0.379660208	0.000157927	5.65E-005
MPP1	-1.397219295	0.379660208	0.000157927	5.65E-005
KMO	-1.242826795	0.422543918	0.009470041	0.008463355
LGALS8	-1.168345299	0.444931363	0.000311396	0.000130224
TTLL4	-1.050093266	0.482936943	0.000406567	0.000180077
CPSF6	-0.999324926	0.500234017	1.58E-005	3.30E-006
THOC6	-0.984099057	0.505541328	0.000509837	0.00023609
A1A5D9_HUMAN	-0.984099057	0.505541328	0.000509837	0.00023609
DNASE1L1	-0.899826348	0.535951238	0.000167797	6.11E-005
C20orf27	-0.712715944	0.610170381	0.001425095	0.000825542
HSPA12B	-0.712715944	0.610170381	0.001425095	0.000825542
HBEGF	0.691760126	1.615252968	1.31E-007	1.03E-008
ADCY4	0.696349179	1.620399089	1.64E-005	3.46E-006
LTB4R	0.696349179	1.620399089	1.64E-005	3.46E-006
RASA4	0.750276956	1.682115718	0.005684707	0.004532202
MCTP1	0.758665871	1.691925297	1.31E-005	2.60E-006
RPH3A	0.758674642	1.691935584	0.000236745	9.29E-005
MTMR11	0.772114221	1.707770632	0.000212991	8.16E-005
PLEKHQ1	0.790712848	1.729929025	7.64E-005	2.32E-005
RASL2_HUMAN	0.815657846	1.760100547	9.64E-007	1.09E-007
STX16	0.816815162	1.761513048	0.000110454	3.65E-005
NPEPL1	0.816815162	1.761513048	0.000110454	3.65E-005
CYP27A1	0.845255204	1.796582522	5.72E-011	1.19E-012
HBG1	0.851121004	1.80390205	0.001271155	0.000716026
HBG2	0.851121004	1.80390205	0.001271155	0.000716026
CALCOCO1	0.876429525	1.835826253	4.61E-005	1.25E-005
TNFAIP2	0.896984091	1.862169105	2.29E-007	1.97E-008
C3AR1	0.923609793	1.896855517	0.004811403	0.0037182
CLMN	0.946959131	1.927805004	0.001774223	0.001084808
FES	0.949791206	1.931593087	5.22E-012	6.81E-014
FBXL5	0.958529448	1.943328034	0.001139583	0.00062446
Q59GI6_HUMAN	0.988782749	1.984509885	0.004772669	0.003679281
NAIP	0.988782749	1.984509885	0.004772669	0.003679281
FCGR1B	0.992864878	1.99013304	1.40E-006	1.70E-007
Q8NAM9_HUMAN	0.992864878	1.99013304	1.40E-006	1.70E-007
SECTM1	1.03837198	2.053908596	0.001131981	0.000619866
LYL1	1.046933552	2.06613361	0.008170965	0.007048387
NFIX	1.046933552	2.06613361	0.008170965	0.007048387
CFD	1.047241556	2.066574761	0.002003684	0.001262019
HLX	1.070954574	2.100822938	1.97E-005	4.31E-006
RASSF4	1.080257483	2.114413415	3.89E-009	1.70E-010
ANPEP	1.150769982	2.220323639	0.00230899	0.001502752
TCF7L2	1.158381111	2.232068203	0.004259753	0.00320629
DYSF	1.178062164	2.262726421	3.57E-009	1.53E-010
HNMT	1.180745094	2.266938251	7.66E-007	8.16E-008
TPPP3	1.30332488	2.467970038	1.50E-010	3.47E-012
GAS7	1.310666477	2.480561072	1.95E-007	1.64E-008
FCGR1A	1.327166805	2.509094495	8.44E-009	4.11E-010
IL8	1.368382696	2.581809753	0.001548739	0.000918202
CENTD3	1.423849247	2.683004078	1.98E-010	4.91E-012

NT5DC2	1.429600865	2.693721808	4.07E-011	7.94E-013
STAB1	1.429600865	2.693721808	4.07E-011	7.94E-013
CFLAR	1.603967505	3.039781245	0.00022804	8.87E-005
FOSB	1.660278381	3.160775088	2.45E-006	3.49E-007
NP_057218.1	1.663527117	3.167900707	5.22E-012	6.78E-014
CDKN1C	1.676213144	3.195879791	0.000146234	5.13E-005
BTG2	1.725791867	3.30761626	0.000493472	0.000227337
CA2	1.764191002	3.39683467	1.32E-008	6.94E-010
CD36	1.921930744	3.789298374	5.40E-010	1.61E-011
SOCS3	1.939878769	3.83673406	1.30E-008	6.82E-010
TMEM176A	2.348791292	5.093972927	7.64E-010	2.44E-011
KLF4	2.812408097	7.024561147	1.58E-009	5.74E-011
CLEC3B	2.965550316	7.811233113	1.54E-014	7.89E-017
CD163	2.992065274	7.95612131	6.36E-012	8.98E-014
KCTD12	3.001816675	8.010080131	8.25E-011	1.80E-012
G0S2	3.025210426	8.141024871	6.06E-007	6.18E-008
CD14	3.088907546	8.508516106	8.47E-015	4.10E-017
EGR1	3.268716046	9.637881386	0.000170455	6.25E-005
CD93	4.225112704	18.70189693	1.47E-020	1.18E-023
