# Supplementary material for: High Expression of Complement Component 5 (C5) at Tumor Site Associates with Superior Survival in Ewing's Sarcoma Family of Tumour Patients
Source: ISRN Oncol. 2011 Oct 2;2011:168712. doi: 10.5402/2011/168712 (PMC3196920; doi:10.5402/2011/168712)
Supplement: Supplementary file 4 [file 168712.f4.pdf]

Gene name	log ratio	fold change	q-value	p-value
MAPK12	-3.357244883	0.097581746640516	4.8219006515766e-18	4.23587373304337e-18
IL15	-2.788195387	0.144766993326912	3.04690449426432e-11	7.84186414542595e-11
CXCL14	-2.527752595	0.173408606080554	4.28469727963729e-09	1.52881530931904e-08
BCL6	-2.083277566	0.235977699709790	1.70076432497995e-11	4.18946746414079e-11
CXCL12	-1.698654293	0.308073331782935	4.81213522777062e-08	2.00880299273869e-07
IL32	-1.485088153	0.357226706580382	0.000167245680174804	0.00110988336363636
INDO	-1.457842471	0.364037134453161	2.76361153518103e-12	6.03910038061413e-12
PGLYRP4	-1.225796966	0.427561256389181	5.25365375960739e-10	1.63202925133624e-09
IL6R	-1.221217176	0.428920692707457	2.58789027797544e-09	8.91712993798201e-09
IL19	-1.131572516	0.456417965027779	3.69670500211022e-11	9.63746920345942e-11
FOXP3	1.111993011	2.16144033288987	6.33788124497605e-14	1.07222215765570e-13
STAT1	1.138997038	2.2022786740609	4.11410128164735e-09	1.46426157405040e-08
MAP4K4	1.181663917	2.26838247693826	2.68553073738761e-11	6.8476692889314e-11
C5	1.18479521	2.27331122771974	2.47732931566637e-08	9.91881334679839e-08
PELI2	1.191117206	2.28329490434862	9.01381182728118e-09	3.37089214658012e-08
IKBKB	1.198995647	2.29579790100733	7.4724466876785e-08	3.21337840877331e-07
IL10RB	1.278394813	2.42568936993011	2.15697771269503e-11	5.42373339531316e-11
IL23A	1.33626986	2.52497632398107	9.77328146786977e-14	1.70445824549574e-13
STAT2	1.33626986	2.52497632398107	9.77328146786977e-14	1.70445824549574e-13
HSPA6	1.352912764	2.55427306313752	8.41955526131707e-08	3.65249379370052e-07
TNFAIP3	1.410810324	2.6588646230428	1.46133276147057e-09	4.85578704041691e-09
MAPK1	1.447421651	2.72720216771854	7.09591129298659e-13	1.40412898639933e-12
IL8	1.499708427	2.82785554956341	1.02455686605556e-07	4.49101602753508e-07
HMGB1	1.584533792	2.99910865771966	8.87250640302558e-13	1.78701478882278e-12
STAT6	1.655704845	3.1507708755422	1.92784086529138e-13	3.51942159589145e-13
SOCS3	1.792509963	3.46417055926045	6.81967782575762e-09	2.50421693129316e-08
SOCS2	1.801297433	3.48533524766671	3.2085641442394e-11	8.2850627724214e-11
MYD88	1.846398163	3.5960128176579	1.07080121504600e-17	9.92861822972462e-18
IRF3	1.888529096	3.70257534966566	3.54300979792916e-14	5.73311251431078e-14
TRAF5	1.98372873	3.95513991038940	4.29134500036905e-17	4.38457919193856e-17
SOCS4	2.052915759	4.14943744803378	7.77801839660649e-15	1.13208056891915e-14
PELI1	2.11679872	4.33730446585109	2.32873206399972e-14	3.65123495269558e-14
ERLIN1	2.235419827	4.70899707773362	5.15596447814792e-16	6.23489183924291e-16
FOS	2.346470055	5.08578352488307	0.000137565298166909	0.000904158099773456
CD14	2.430671583	5.39144347066627	6.04263814341399e-15	8.62365124205907e-15
PGLYRP2	2.58900383	6.01683097882971	9.29222413880672e-15	1.37003582812466e-14
MAPK7	2.790153388	6.91703323192069	7.33034831209276e-17	7.76673099008104e-17
JAK1	2.960934031	7.78627894787052	7.06020504907908e-24	2.40570258204756e-24
CXCR4	3.700324777	12.9989643173022	9.52464926338294e-22	4.67230064918968e-22
MIF	3.843152975	14.3517322436330	1.01276129258595e-32	5.59196084217635e-34
LY96	4.464987403	22.0848848219049	1.25504381540421e-27	2.07267409774336e-28
SPP1	4.823136485	28.307971863345	9.27605994912501e-19	7.30429845089267e-19
